# Expression and Activation of BK_Ca_ Channels in Mice Protects Against Ischemia-Reperfusion Injury of Isolated Hearts by Modulating Mitochondrial Function

**DOI:** 10.3389/fcvm.2018.00194

**Published:** 2019-01-28

**Authors:** Sumanta Kumar Goswami, Devasena Ponnalagu, Ahmed T. Hussain, Kajol Shah, Priyanka Karekar, Shubha Gururaja Rao, Andrea L. Meredith, Mahmood Khan, Harpreet Singh

**Affiliations:** ^1^Department of Pharmacology and Physiology, Drexel University College of Medicine, Philadelphia, PA, United States; ^2^Department of Physiology and Cell Biology, Wexner Medical Center, Ohio State University, Columbus, OH, United States; ^3^Department of Physiology, University of Maryland, Baltimore, MD, United States; ^4^Department of Emergency Medicine, Wexner Medical Center, Ohio State University, Columbus, OH, United States

**Keywords:** cardiac mitochondria, BK_Ca_ channels, reactive oxygen species, ischemia-reperfusion injury, myocardial infarction, ischemic preconditioning

## Abstract

**Aims:** Activation and expression of large conductance calcium and voltage-activated potassium channel (BK_Ca_) by pharmacological agents have been implicated in cardioprotection from ischemia-reperfusion (IR) injury possibly by regulating mitochondrial function. Given the non-specific effects of pharmacological agents, it is not clear whether activation of BK_Ca_ is critical to cardioprotection. In this study, we aimed to decipher the mechanistic role of BK_Ca_ in cardioprotection from IR injury by genetically activating BK_Ca_ channels.

**Methods and Results:** Hearts from adult (3 months old) wild-type mice (C57/BL6) and mice expressing genetically activated BK_Ca_ (Tg-BK_Ca_^R207Q^, referred as Tg-BK_Ca_) along with wild-type BK_Ca_ were subjected to 20 min of ischemia and 30 min of reperfusion with or without ischemic preconditioning (IPC, 2 times for 2.5 min interval each). Left ventricular developed pressure (LVDP) was recorded using Millar's Mikrotip® catheter connected to ADInstrument data acquisition system. Myocardial infarction was quantified by 2,3,5-triphenyl tetrazolium chloride (TTC) staining. Our results demonstrated that Tg-BK_Ca_ mice are protected from IR injury, and BK_Ca_ also contributes to IPC-mediated cardioprotection. Cardiac function parameters were also measured by echocardiography and no differences were observed in left ventricular ejection fraction, fractional shortening and aortic velocities. Amplex Red® was used to assess reactive oxygen species (ROS) production in isolated mitochondria by spectrofluorometry. We found that genetic activation of BK_Ca_ reduces ROS after IR stress. Adult cardiomyocytes and mitochondria from Tg-BK_Ca_ mice were isolated and labeled with Anti-BK_Ca_ antibodies. Images acquired via confocal microscopy revealed localization of cardiac BK_Ca_ in the mitochondria.

**Conclusions:** Activation of BK_Ca_ is essential for recovery of cardiac function after IR injury and is likely a factor in IPC mediated cardioprotection. Genetic activation of BK_Ca_ reduces ROS produced by complex I and complex II/III in Tg-BK_Ca_ mice after IR, and IPC further decreases it. These results implicate BK_Ca_-mediated cardioprotection, in part, by reducing mitochondrial ROS production. Localization of Tg-BK_Ca_ in adult cardiomyocytes of transgenic mice was similar to BK_Ca_ in wild-type mice.

## Introduction

The large conductance calcium and voltage-activated potassium channels (MaxiK, BK_Ca_, K_Ca_1.1) encoded by *Kcnma1* gene are ubiquitously expressed in excitable and non-excitable cells (1, 2). The functional channel is comprised of four pore-forming α-subunits, each with seven transmembrane domains where S4 serves as a voltage sensor and C-terminus contains Ca^2+^-sensing RCK1 and RCK2 domains (3). Ca^2+^ and voltage sensing allow activation of BK_Ca_ (4), resulting in its physiological involvement in neurotransmitter release and secretion (2). Increasing evidence indicates that BK_Ca_ channels are located in intracellular organelles in addition to the plasma membrane, extending their functional roles in cellular physiology from organelle to organ level (1, 2, 5–10).

Studies involving activation (10–15) and inactivation (11, 16) with pharmacological and genetic tools, including global (10), and tissue-specific knockouts (17), have implicated BK_Ca_ channels in cardiac function, neuroprotection (18), and cardioprotection from ischemia-reperfusion (IR) injury, in addition to IR-induced inflammation and mucosal barrier disruption in the small intestine (19). Further, it was shown that BK_Ca_ is present in the mitochondria of adult cardiomyocytes (10, 20). Tissue-specific knockouts in which BK_Ca_ was ablated in adult cardiomyocytes showed that expression of mitochondrial BK_Ca_ is responsible for its cardioprotective effect (17). It has been shown that agonists or antagonists have no effect on global (10) and cardiomyocytes-specific (17) knockouts. However, mice expressing activated BK_Ca_ have not been tested for cardioprotection from IR injury (8). Genetically modifying BK_Ca_ in mice by introducing a mutation responsible for its constitutive activation (8), independent of pharmacological agents, can further support the role of BK_Ca_ in cardioprotection from IR injury.

One of the possible outcomes of pharmacological activation or inactivation of BK_Ca_ is decrease/increase in the production of reactive oxygen species (ROS) (21–24). The reduction in the levels of ROS accompanied by “mild” mitochondrial uncoupling (25) by BK_Ca_ agonists is assigned as a possible mechanism for organ and cellular protection from IR injury (26). As stated earlier, all of these studies rely on the use of pharmacological tools with possible non-specific effects. To understand the role of activation of BK_Ca_ and its influence on mitochondrial ROS generation, studies need to be performed independent of the pharmacological agents. Non-specific and off-target effects of pharmacological tools have generated reservations (12) on the role of BK_Ca_ in modulating levels of mitochondrial ROS as well as cardioprotection from IR injury.

In the current study, we have used genetically-activated mice where BK_Ca_ is constitutively active due to incorporation of a gain of function mutation (Tg-BK^R207Q^ or Tg-BK_Ca_) (8) to test the role of BK_Ca_ activation in mitochondrial ROS generation and cardioprotection from IR injury. We have established that the activation of BK_Ca_ is vital for a cardioprotective effect in both IR as well as IPC using an *ex vivo* isolated perfused heart model. We have further shown that activation of BK_Ca_, attenuates ROS from complex I and complex II/III of mitochondria only after IR injury. Our results presented here further corroborate the role of BK_Ca_ in cardioprotection.

## Methods

All of the experiments on mice were approved by the Institutional Animal Care and Use Committee at the Drexel University and the Ohio State University. Animals were housed in the vivarium with food and water available *ad libitum*. Experiments were carried out on 3 month-old male and female. Experimentalists were blinded for the genotype of mice used.

### Materials

Horseradish peroxidase (Sigma-Aldrich # P6782), DC^TM^ protein assay kit (BIO-RAD Laboratories, #500-0113, 500-0114, 500-0115), glutamate (Sigma-Aldrich # G1626), malate (Sigma-Aldrich # M6773), succinate (Fluka # 14160), pyruvate (Sigma-Aldrich # P2256), Amplex® Red (Invitrogen/Thermo Fisher Scientific # A12222), anti-BK_Ca_ antibody (Alomone labs, APC-21 lot #5) were procured for the study.

### Ischemia-Reperfusion Injury Model *ex vivo*

Wild-type mice or mice co-expressing genetically activated BK_Ca_ (Tg-BK_Ca_) (8) were anesthetized with 87 mg/kg of ketamine and 13 mg/kg of xylazine by administering these agents intraperitoneally (i.p). The hearts were rapidly excised, washed in ice-cold modified Krebs-Henseleit (KH, pH 7.4, concentrations in mM: 118 NaCl, 4.7 KCl, 1.2 KH_2_PO_4_, 1.2 MgSO_4_, 24 NaHCO_3_, 11.1 Glucose, 2 CaCl_2_, 1 sodium pyruvate) solution, mounted on a cannula and perfused with KH solution at 37°C at a constant volume (2 mL/min). A pressure transducer (Millar Mikrotip® catheter) was introduced to the left ventricle, and after achieving a stable baseline, hearts were subjected to 20 min of global ischemia and 30 min of reperfusion (27). A subgroup of hearts was subjected to IPC before IR to evaluate the role of BK_Ca_ in IPC-mediated cardioprotection. Two sets of 2.5 min of ischemia and 2.5 min of reperfusion were used to precondition hearts before IR. Left ventricular developed pressure (LVDP) was recorded using Powerlab® hardware data acquisition system along with LabChart data acquisition and analysis software (ADInstrument, USA). After recording cardiac function, hearts either were analyzed for myocardial infarction by TTC staining or used to rapidly isolate mitochondria and measure ROS. WT IR, WT IPC, Tg-BK_Ca_ IR, and Tg-BK_Ca_ IPC group had 7, 8, 6, and 7 mice, respectively, for studying cardiac function and myocardial infarction in section- Measurement of Myocardial Infarction.

### Measurement of Myocardial Infarction

Isolated hearts were thawed, cut into 5 horizontal 2-mm sections using a heart slicer matrix, and incubated for 20 min at room temperature in 2% (*w/v*) TTC solution in phosphate buffer saline at a pH of 7.4. Images were obtained using Nikon SMZ1000 microscope connected to Nikon digital sight DS-Fi2 camera and analyzed with Image J. WT IR, WT IPC, Tg-BK_Ca_ IR, and Tg-BK_Ca_ IPC group had 7, 8, 6, and 7 mice, respectively.

### Measurement of Cardiac Function by Echocardiography

To ensure that the results obtained related to cardiovascular function and the myocardial infarction is not due to the altered cardiac function of transgenic mice, we recorded the echocardiograph of WT (*n* = 6) and Tg-BK_Ca_ (*n* = 5) mice before they were used for IR or IPC study. Vevo2100® imaging system (FUJIFILM VisualSonics) with MS400 probe was used to acquire images (28). Briefly, mice were anesthetized using 2% (*v/v*) isoflurane in carbogen(95% oxygen and 5% carbon dioxide) with heart rate was maintained at more than 450 bpm. Cardiac functions were measured in both B and M mode with the probe positioned in long axis. Mean and peak velocities of the ascending and descending aorta were also recorded using color Doppler. Images were analyzed using Vevo Lab 3.1.1 analysis software.

### Measurement of ROS

In case of some hearts, after reperfusion for 10 min, the cardiac tissue was rapidly cut into pieces in 2 mL of ice-cold mitochondrial isolation buffer A (sucrose 70 mM, mannitol 210 mM, EDTA 1 mM, Tris HCl 50 mM, pH 7.4) and homogenized using a hand-held glass homogenizer without using any detergent. The homogenates were centrifuged at 4°C, 2,500 g for 5 min and supernatants were collected and centrifuged at 4°C, 12,000 g for 10 min. The mitochondrial pellets were resuspended in 100 μL of mitochondria isolation buffer B (sucrose 70 mM, mannitol 210 mM, EDTA 0.1 mM, Tris HCl 50 mM, pH 7.4) and again centrifuged at 4°C, 12,500 g for 5 min. The pellets were again resuspended in 100 μL of ROS buffer (EGTA 1mM, EDTA 1mM, Tris HCl 20 mM, sucrose 250 mM, pH 7.4, 0.15% BSA was added before use) and centrifuged at 4°C, 12,000 g for 5 min. The pellets were then resuspended in 55 μL of ROS buffer and stored on ice until used for quantifying the generation of ROS. Horse Radish Peroxidase (0.5 μL of 10 mg/mL in 0.1M phosphate buffer, pH 6) solutions were added to 2 mL of ROS buffer in the cuvette and the solutions were continuously stirred with a magnetic stirrer at 37°C. Basal absorbance was recorded at 560 nm excitation and 590 nm emission wavelength using Hitachi F-2710 fluorescence spectrophotometer. After 1 min, 2 μL of 10 μM amplex red was added to the cuvette. After another 1 min, 25 μL of the mitochondrial suspension was added to the cuvette. Subsequently, mitochondrial substrates, either glutamate (5 mM) and malate (5 mM) (WT IR, *n* = 3; WT IPC, *n* = 4; Tg-BK_Ca_ IR, *n* = 4; Tg-BK_Ca_ IPC, *n* = 4) or succinate (3 mM) (WT IR, *n* = 3; WT IPC, *n* = 4; Tg-BK_Ca_ IR, *n* = 4; Tg-BK_Ca_ IPC, *n* = 4), were added after 90 s and absorbance was recorded for a total duration of 45 min. Glutamate and malate are substrates for complex I mediated ROS production, and succinate as a substrate for complex II result in ROS production from complex III and by backflow of electrons to complex I. ROS generated was normalized per μg of mitochondrial protein. The remaining mitochondrial samples were used for measuring the protein concentration using DC^TM^ protein assay kit from BIO-RAD Laboratories, Inc. and SPECTRAmax® spectrometer from Molecular Devices. The level of ROS produced was recorded using FL solutions software (Hitachi, USA) and ROS produced in arbitrary fluorescence units (*a.u*.) per μg of mitochondria were measured (29). For baseline ROS measurements, hearts from WT (*n* = 3) and Tg-BK_Ca_ mice (*n* = 3) were used without exposing the hearts to IR and IPC.

### Visualization of BK_ca_

Adult mice cardiomyocytes were isolated and loaded with mitotracker (100 nM, excitation: 579 nm and emission: 599 nm) for 60 min at 4°C followed by fixation with 4% paraformaldehyde. Cardiomyocytes loaded with mitotracker (*n* = 5) were labeled with anti-BK_Ca_ antibodies (Alomone labs, APC21, 1:200) to study the localization of BK_Ca_ in mitochondria. For studying the localization of BK_Ca_ on plasma membrane, cardiomyocytes labeled with anti-BK_Ca_ antibodies were also marked with plasma membrane marker, wheat germ agglutinin conjugated with Alexa Fluor 488 (WGA, 1:1,000, *n* = 5) as described earlier (10). Isolated cardiac mitochondria from wild-type (*n* = 6), Tg-BK_Ca_ (*n* = 5), and *Kcnma1*^−/−^ (*n* = 5) mice were loaded with mitotracker and labeled with anti-BK_Ca_ antibodies as described earlier (10). Atto647N anti-rabbit secondary antibody was used to label BK_Ca_. Images were acquired using a confocal microscope (FV1000, Olympus) and median filtered (29).

### Statistical Analysis

Student's *t*-test (unpaired and one-tailed) and one-way ANOVA followed by Tukey's multiple comparison tests were used to measure the statistical difference between groups. Values are presented as mean ± SEM of 3–8 observations. A value of *p* < 0.05 was considered to be statistically significant.

## Results

Overall, our results demonstrate that expression and activation of BK_Ca_ are vital for cardioprotection from IR injury as well as IPC-mediated cardioprotection. Cardioprotection mediated by BK_Ca_ is possibly modulated by mitochondrial ROS production.

### Genetic Activation of BK_Ca_ Preserves Cardiac Function Recovery During Reperfusion

**Figure 1:**
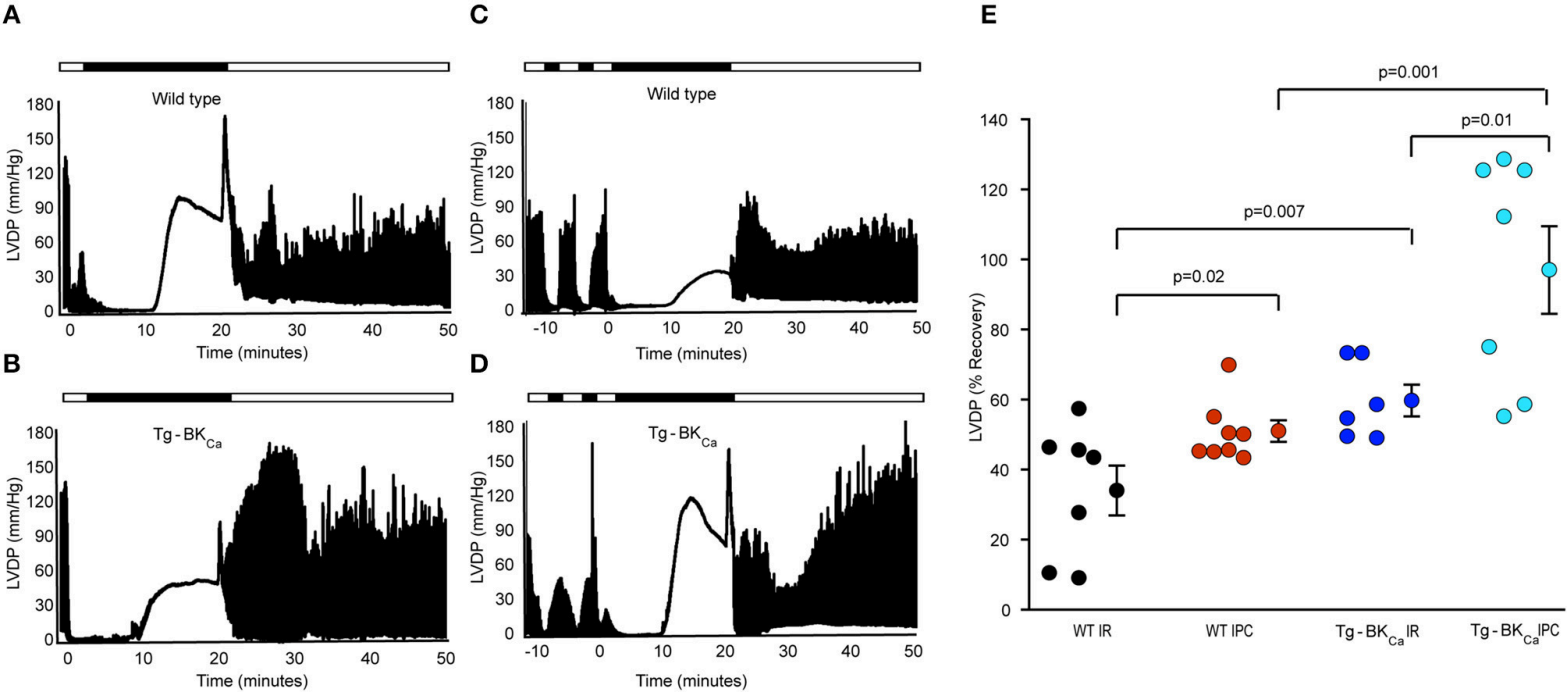
Activation of BK_Ca_ and IPC promotes the recovery of cardiovascular function from IR-injury. LVDP traces of heart of WT animal subjected to IR **(A)** and IPC **(C)** along with the LVDP traces of the heart of Tg-BK_Ca_ animal subjected to IR **(B)** and IPC **(D)** are shown. LDVP of WT hearts subjected to IPC (*n* = 8) showed improved recovery, in comparison to IR (*n* = 7) **(A,C,E)**. LDVP of Tg-BK_Ca_ hearts subjected to IPC (*n* = 7), in comparison to IR (*n* = 6) also recovered better **(B,D,E)**. LDVP of Tg-BK_Ca_ hearts (*n* = 6) subjected to IR exhibited improved recovery, in comparison to WT (*n* = 7) hearts **(A,B,E)**. IPC promoted recovery of hearts from Tg-BK_Ca_ mice (*n* = 7) better than WT mice (*n* = 8) **(C,D,E)**. The average baseline values of LVDP was 80 mmHg for 28 animals from 4 different groups. Baseline LVEDP ranged from 5 to 10 mmHg. The contractures during ischemia and reperfusion were seen in few hearts from each group in this study. The ischemic contractions are dependent on depletion of ATP during ischemia and reperfusion-induced increase in the cytosolic calcium ion. **(E)** Quantification of percentage recovery of LVDP as shown in **(A–D)**, black circles indicate WT subjected to IR; red, WT IPC; blue, TG-BK_Ca_ IR and light blue Tg-BK_Ca_ IPC. The values are presented as mean ± SEM of 6–8 readings.

The role of activation of BK_Ca_ in cardioprotection has been demonstrated by usage of pharmacological tools (1, 10, 11, 13, 14, 30–32), and these drugs are known to have non-specific effects (12). Even though the expression of BK_Ca_ is vital for cardioprotection from IR injury (10, 17), the role of activation of BK_Ca_ in cardioprotection is not yet well-characterized. We have utilized Tg-BK_Ca_ mice expressing genetically activated BK_Ca_ in a DEC splice variant background (GenBank: JX429072.1) (8) and carried out IR injury assays with and without IPC (Figures 1A–D). Tg-BK_Ca_ mice are viable, normal in body weight (8), and exhibit increased BK_Ca_ protein expression in a wide variety of tissues as well as display increased BK_Ca_ channel currents (8). Tg-BK_Ca_ is under *Period1 (Per1)* promoter which is ubiquitously expressed in all tissues. Tg-BK_Ca_ mice express the R207Q mutation in the S4 voltage sensor of the BK_Ca_ α subunit which strongly augments voltage-dependent gating of the channel without affecting the Ca^2+^-dependent activation (33). In the Tg-BK_Ca_ mice, cardiac functional recovery was higher in comparison to the wild-type control (Figures 1B vs. A). Percentage recovery of LVDP after IR-injury was significantly higher (*p* = 0.02) for Tg-BK_Ca_ mice (60 ± 5%, *n* = 6), in comparison to WT mice (34 ± 7%, *n* = 7) (Figures 1A,B,E).

### Genetic Activation of BK_Ca_ Confers Protection During Ischemic Preconditioning

In addition to protecting the heart from IR injury, pharmacological activation or blocking of BK_Ca_ has also been implicated in mediating cardioprotection through IPC (17, 32, 34). We have also tested whether activation of BK_Ca_ plays a role in cardioprotection mediated by IPC. Two brief IPC events before IR provided cardioprotection to wild-type (Figures 1A,C,E) as well as Tg-BK_Ca_ mice (Figures 1B,D,E). The percentage recovery of LVDP seen with the hearts from Tg-BK_Ca_ mice exposed to IPC (*n* = 7) was significantly higher than in Tg-BK_Ca_ mice hearts exposed to IR (*p* = 0.01, *n* = 6) and in WT mice hearts exposed to IPC (*p* = 0.001, *n* = 8). Percentage recovery of LVDP at the end of reperfusion was 51 ± 3% (*n* = 8) and 97 ± 12% (*n* = 7) for WT IPC and Tg-BK_Ca_ IPC groups, respectively.

### Genetic Activation of BK_Ca_ Attenuates Myocardial Infarction Following IR

**Figure 2:**
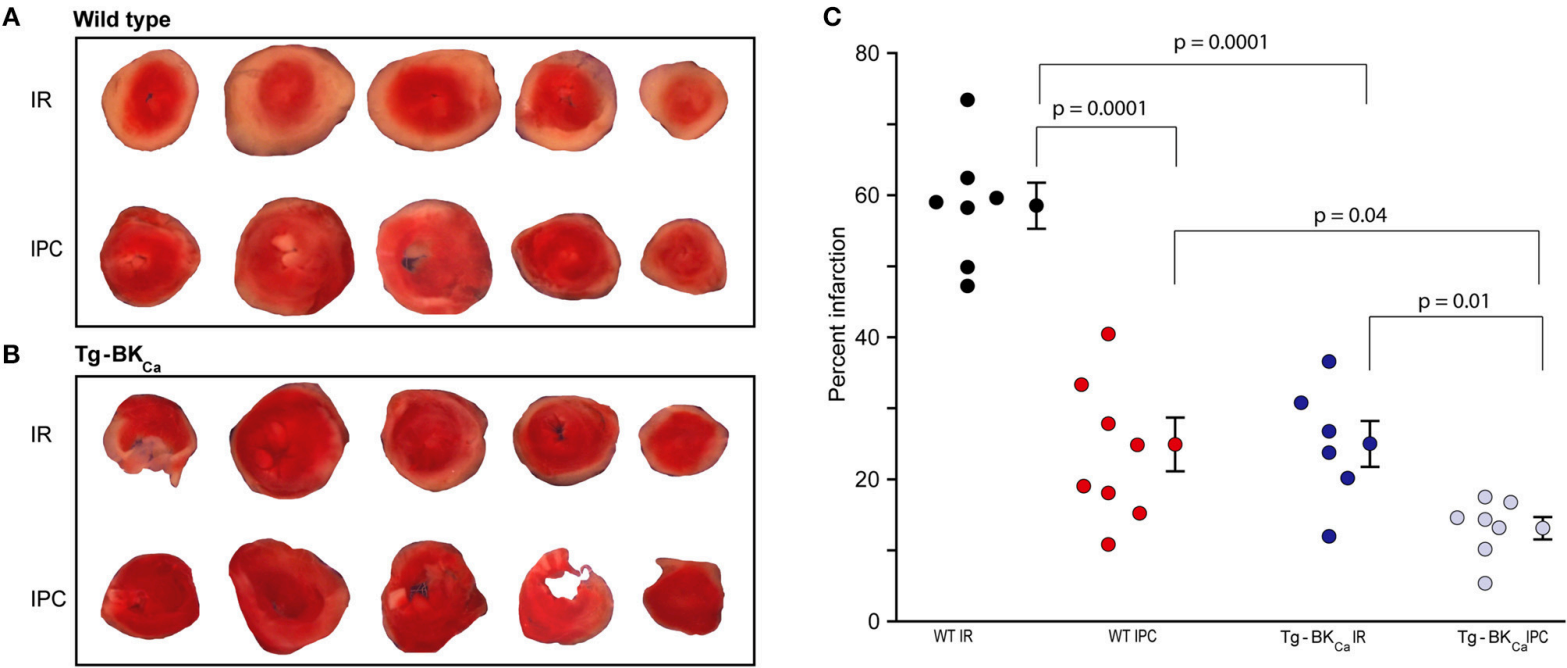
Activation of BK_Ca_ and IPC protect hearts from IR-injury. TTC staining differentiated between infarcted (white) and viable cells (red) in the hearts of WT **(A)** and Tg-BK_Ca_ **(B)** mice. **(C)** The infarcted area was quantified by planimetry using ImageJ. Quantification of **(A,B)**, black circles indicate WT subjected to IR; red, WT IPC; blue, TG-BK_Ca_ IR and light blue Tg-BK_Ca_ IPC. Hearts of WT mice subjected to IPC (*n* = 8) presented less infarction as compared to hearts subjected to only IR (*n* = 7) **(A,C)**. Similarly, the hearts of Tg-BK_Ca_ mice subjected to IPC (*n* = 7) were protected better than hearts subjected to only IR (*n* = 6) **(B,C)**. The hearts from Tg-BK_Ca_ mice were better protected from WT mice when subjected to IR and IPC **(A–C)**. Values are presented as mean ± SEM of 6–8 readings.

The degree of myocardial infarction was quantified following IR using TTC staining. Viable cells appeared red while dead cells appeared pale yellow (Figure 2). The degree of infarction was higher in WT IR group (*n* = 7) with 59 ± 3% cell death but infarction was significantly less in (*p* = 0.0001) in WT IPC group (*n* = 8) with only 25 ± 4% cell death (Figures 2A–C). Hearts from Tg-BK_Ca_ mice sustained less infarction [25 ± 3% (*n* = 6), *p* = 0.04] after IR in comparison to hearts from WT mice [59 ± 3% (*n* = 7)] (Figures 2A–C, *p* = 0.0001). IPC further protected myocardium of the Tg-BK_Ca_ (*n* = 7) mice which exhibited the least infarction to the heart cells (13 ± 2%), (Figures 2A–C).

Taken together, our results (Figures 1, 2) implicate genetic activation of BK_Ca_ in cardioprotection from IR injury as well as cardioprotection mediated by IPC.

### Cardiac Functional Parameters of WT and Tg-BK_Ca_ Mice Did Not Alter at the Baseline

**Figure 3:**
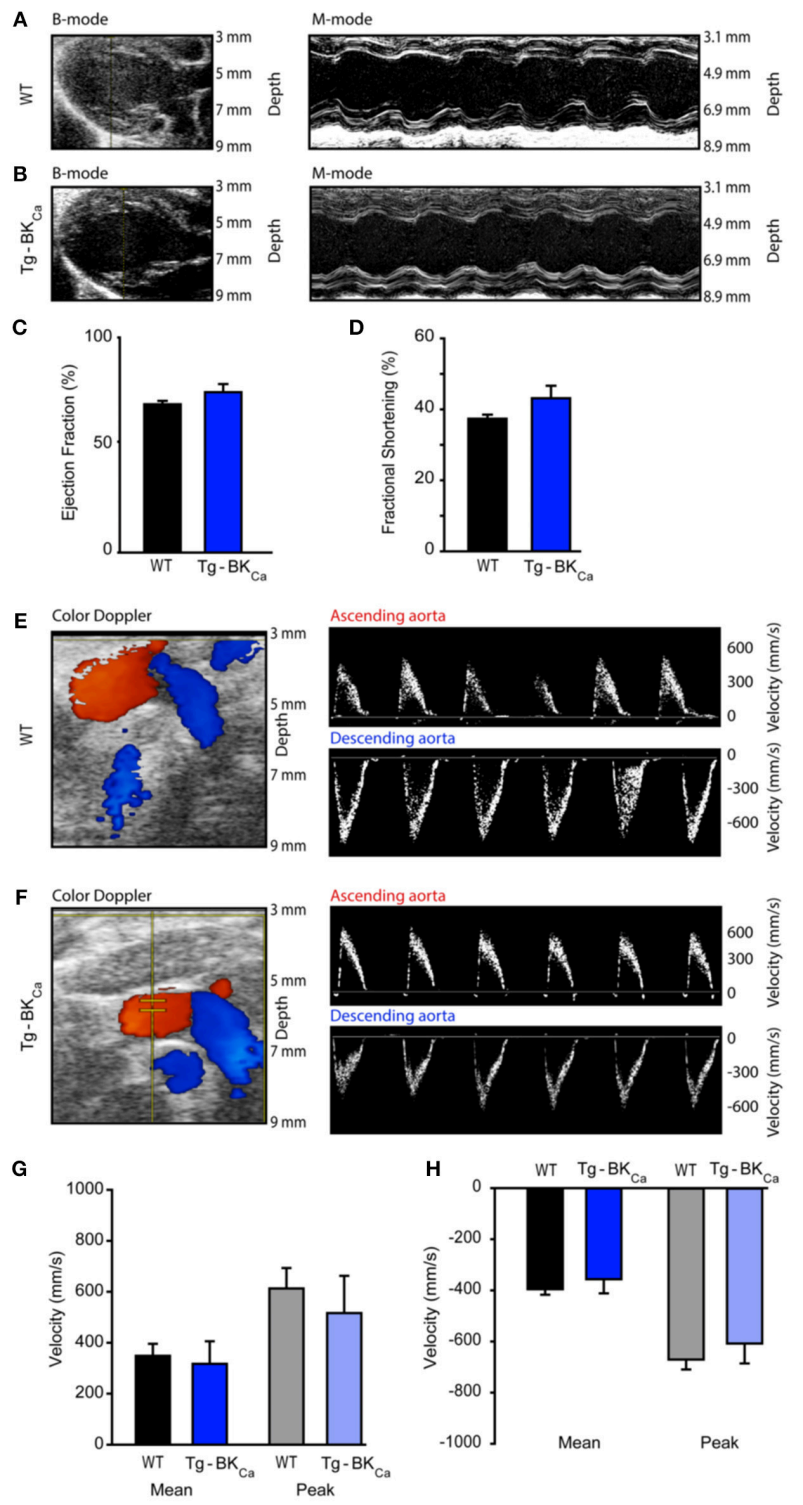
Cardiovascular function of WT and Tg-BK_Ca_ mice. WT and Tg-BK_Ca_ mice were anesthetized and comprehensive echocardiography measurements were obtained to evaluate cardiac function. **(A,B)** Representative B-mode and M-mode of mice hearts from both groups. Left ventricular ejection fraction **(C)** and fractional shortening **(D)** were measured using the parasternal long axis view. There was no difference between WT (black) and Tg-BK_Ca_ (blue) mice. **(E,F)** Color Doppler of ascending (red) and descending (blue) aorta from both groups. The right panel shows representative images of peak velocities of ascending and descending aorta from both groups. **(G)** Mean (black and blue) and peak (gray and light blue) velocities of ascending aorta of WT and Tg-BK_Ca_ mice showed no difference. **(H)** Mean (black and blue) and peak (gray and light blue) velocities of descending aorta of WT and Tg-BK_Ca_ mice showed no difference. The values are presented as mean ± SEM of 5–6 readings.

Left ventricular ejection fraction (LVEF) and fractional shortening (LVFS) assessed by echocardiography (Figures 3A,B) did not demonstrate any differences. The LVEF of WT and Tg-BK_Ca_ were 69 ± 2% (*n* = 6) and 75 ± 4% (*n* = 5), respectively (Figure 3C). The fractional shortening of WT and Tg-BK_Ca_ were 37 ± 1% (*n* = 6) and 43 ± 4% (*n* = 5), respectively (Figure 3D). Similarly, we did not observe any difference in the mean and peak velocities in the ascending aorta and descending aorta of both the WT and Tg-BK_Ca_ mice (Figures 3E–H). Mean ascending aorta velocities of WT and Tg-BK_Ca_ were 347 ± 47 mm/s (*n* = 6) and 315 ± 88 mm/s (*n* = 5), respectively (Figure 3G). The peak ascending aorta velocities of WT and Tg-BK_Ca_ were 611 ± 80 and 515 ± 145 mm/s, respectively (Figure 3G). The mean descending aorta velocities of WT and Tg-BK_Ca_ were −398 ± 22 mm/s (*n* = 6) and −358 ± 56 mm/s (*n* = 5), respectively (Figure 3H). The peak descending aorta velocities of WT and Tg-BK_Ca_ were −661 ± 38 mm/s (*n* = 6) and −616 ± 79 mm/s (*n* = 5), respectively (Figure 3H).

### Genetic Activation of BK_Ca_ Reduces the Production of Mitochondrial ROS

**Figure 4:**
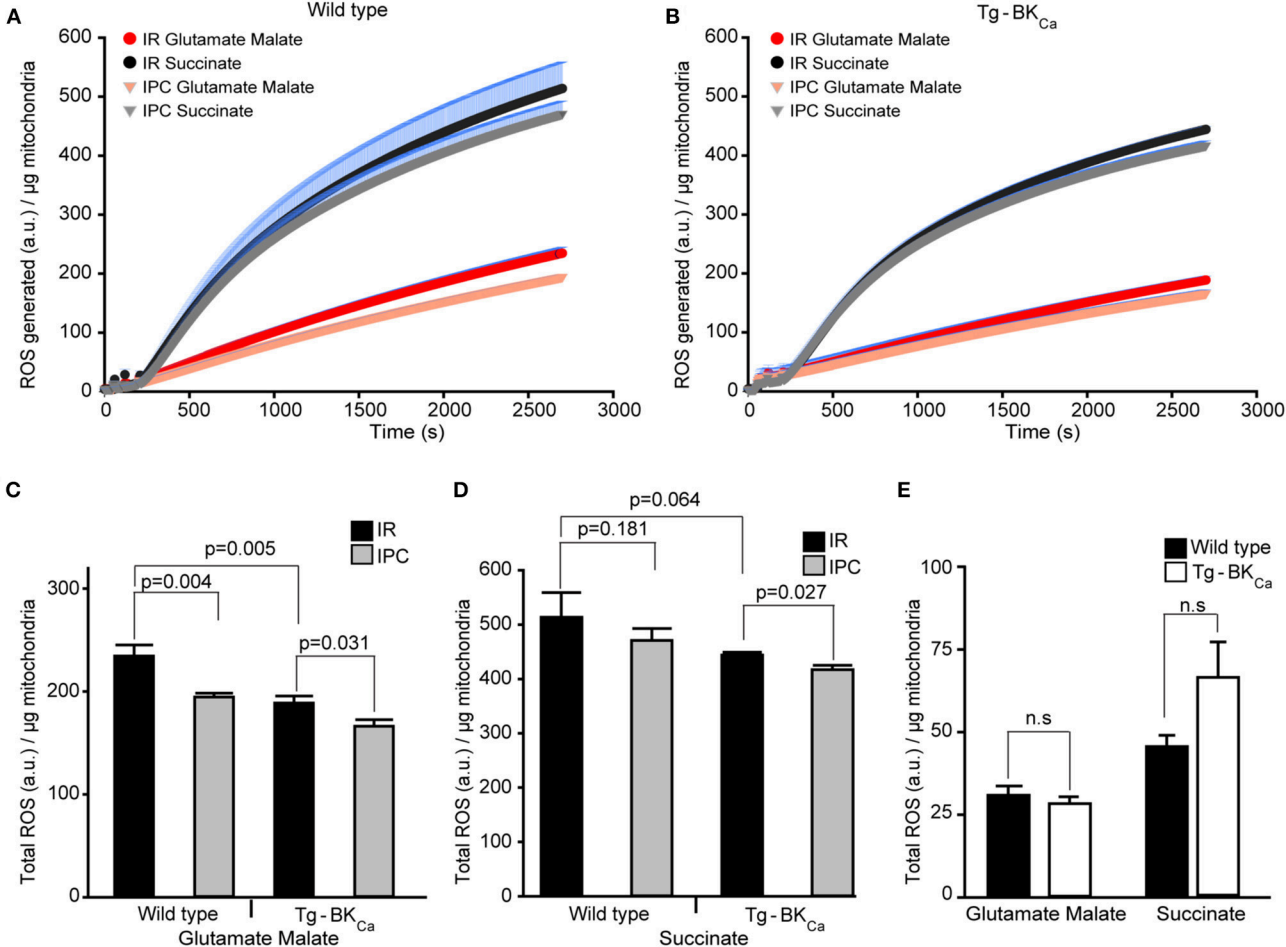
Cardioprotection is inversely proportional to the amount of ROS produced during reperfusion. ROS produced by the mitochondria from the heart of WT **(A)** or Tg-BK_Ca_ **(B)** mice exposed to IR with or without IPC. The generation of ROS by succinate (black and gray) is higher than glutamate and malate (red and orange). **(C)** Quantification of total ROS produced with glutamate and malate as substrate. ROS produced by mitochondria from the heart of WT mice exposed to IR (*n* = 3) is higher, in comparison to heart exposed to IPC (*n* = 4). Similarly, ROS produced by mitochondria from the heart of Tg-BK_Ca_ mice exposed to IR (*n* = 4) is higher, in comparison to heart exposed to IPC (*n* = 4). ROS produced with glutamate and malate by mitochondria from the heart of WT mice exposed to IR is higher than Tg-BK_Ca_ heart exposed to IR. **(D)** Quantification of total ROS produced with succinate as substrate. Mitochondrial ROS from the heart of WT mice exposed to IR (*n* = 3) is not different, in comparison to heart exposed to IPC (*n* = 4). However, ROS produced by cardiac mitochondria isolated from the heart of Tg-BK_Ca_ mice exposed to IR (*n* = 4) is higher than heart exposed to IPC (*n* = 4). **(E)** The amount of ROS produced by the mitochondria isolated from the heart of BK_Ca_ and Tg-BK_Ca_ mice not subjected to IR are not different in presence of any substrate. Mitochondrial protein yield was measured, and it was between 0.3 and 0.6 μg/μL. ROS accumulated / μg of mitochondrial protein was expressed for 45min continuously in **(A,B)**. ROS produced a.u./μg of mitochondrial protein was expressed for 45th min in **(C–E)**. Values are presented as mean ± SEM of 3–4 independent experiments.

ROS produced during IR-mediated injury is well-characterized in cardiac cell death (35), and BK_Ca_ has been pharmacologically implicated in the modulation of cardiac mitochondrial ROS generation (17, 19, 21, 22, 24, 26, 32, 36, 37). Given that BK_Ca_ is present exclusively in mitochondria of adult cardiomyocytes (10) and plays a direct role in cardioprotection from IR injury (17), we tested whether activation of BK_Ca_ can directly modulate mitochondrial ROS production. We quantified the amount of ROS produced by isolated mitochondria after 20 min of ischemia and 10 min of reperfusion (Figures 4A,B*)*. With IPC (*n* = 4), the amount of ROS produced with glutamate and malate as substrates for mitochondria from hearts of WT mice were significantly (*p* = 0.004) reduced, in comparison to hearts only exposed to IR (*n* = 3) (234 ± 10 *a.u*./μg of mitochondria vs. 193 ± 4 *a.u*./μg of mitochondria, Figure 4C). The amount of ROS produced after IPC (*n* = 4) with glutamate and malate as substrates from the heart of Tg-BK_Ca_ mice was also significantly (*p* = 0.003) reduced, in comparison to hearts only exposed to IR (*n* = 4) (188 ± 7 *a.u*./μg of mitochondria vs. 166 ± 6 *a.u*./μg of mitochondria, Figure 4C). Surprisingly, ROS produced from mitochondria isolated from hearts of Tg-BK_Ca_ mice subjected to IR showed similar levels of ROS as wild-type subjected to IPC (Figure 4C).

The ROS produced by succinate as a substrate for cardiac mitochondria isolated from Tg-BK_Ca_ mice exposed to IPC (*n* = 4) was significantly (*p* = 0.027) decreased, in comparison to hearts exposed to IR (*n* = 4) (444 ± 6 *a.u*./μg of mitochondria vs. 417 ± 8 *a.u*./μg of mitochondria, Figures 4B,D*)*. However, the ROS produced by succinate from the heart of WT mice exposed to IPC (*n* = 4) was similar (*p* = 0.181) to WT mice hearts exposed to IR (*n* = 3) alone (514 ± 45 *a.u*./μg of mitochondria vs. 471 ± 21 *a.u*./μg of mitochondria, Figures 4B,D). These results indicate that IPC decreases ROS levels from complex I (Figure 4C) with glutamate and malate as a substrate, and not from complex II/III (Figure 4D) where succinate was used as a substrate in wild-type mice. ROS produced in presence of glutamate and malate, or succinate was lower in Tg-BK_Ca_ mice (Figures 4C,D).

We further measured ROS levels in mitochondria rapidly (29) isolated from hearts of wild-type (*n* = 3) and Tg-BK_Ca_ mice (*n* = 3) which were not subjected to any ischemic stress. Surprisingly, there were no differences observed in ROS produced by mitochondria in the presence of glutamate-malate or succinate (Figure 4E). Although the ROS produced by succinate in Tg-BK_Ca_ mice was ~30% higher, in comparison to WT mice, we did not see any statistical difference due to wide variation in the level of ROS even with 95% power. The cardioprotective effect shown here using transgenic Tg-BK_Ca_ mice is similar to that of pharmacological preconditioning using activators of BK_Ca_ (38, 39).

### BK_Ca_ Co-localizes to Mitochondria of Mice Cardiomyocytes

**Figure 5:**
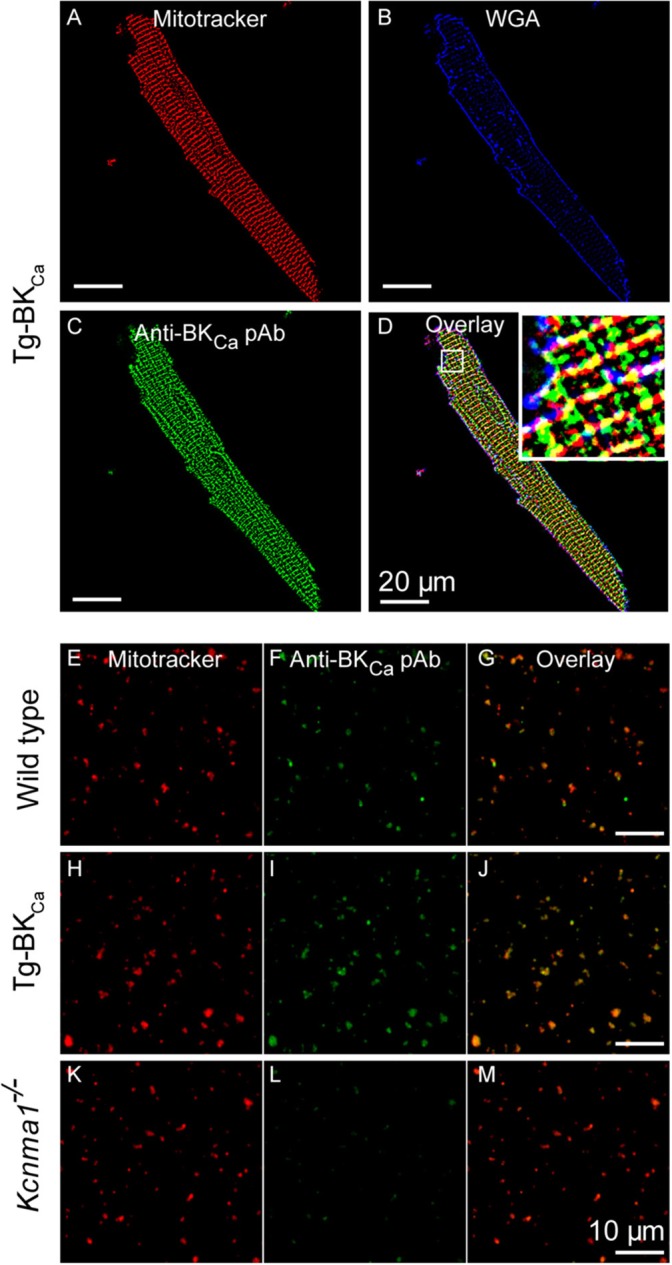
BK_Ca_ and Tg-BK_Ca_ localize in the mitochondria of heart. We isolated the cardiomyocytes by enzymatic digestion from the heart of Tg-BK_Ca_ animals using Langendorff apparatus. The cardiomyocytes were fixed and labeled with mitochondrial marker (MitoTracker^TM^) **(A)** plasma membrane marker (wheat germ agglutinin/WGA) **(B)** and BK_Ca_ marker **(C)**. Overlay of these images **(D)** confirmed that Tg-$BK_{\text{Ca}}{}^{\text{R207Q}}$ are found to be localized in the mitochondria. Mitochondria were isolated from the cardiomyocytes of WT mice and labeled with mitotracker **(E)** and Anti-BK_Ca_ polyclonal antibody **(F)**. Overlay of both the images **(G)** showed that BK_Ca_ is present in the mitochondria. Similarly, mitochondria were isolated from the cardiomyocytes of Tg-BK_Ca_ mice and labeled with mitotracker **(H)** and Anti-BK_Ca_ polyclonal antibody **(I)**. Overlay of both the images **(J)** showed that Tg-$BK_{\text{Ca}}{}^{\text{R207Q}}$ is present in the mitochondria. Mitochondria were also isolated from the cardiomyocytes of *Kcnma1*^−/−^ mice, and labeled with mitotracker **(K)** and Anti-BK_Ca_ polyclonal antibody **(L)**. Overlay of both the images **(M)** showed that BK_Ca_ was effectively deleted in the mice (*n* = 5).

As stated earlier, in adult cardiomyocytes BK_Ca_ has been exclusively localized to the inner membrane of mitochondria (10, 20). Our results implicate BK_Ca_ in mitochondrial ROS generation under ischemic stress. We tested whether BK_Ca_ localizes to cardiac mitochondria of Tg-BK_Ca_ mice. In adult cardiomyocytes, BK_Ca_ localizes to mitochondria loaded with mitotracker but not in the plasma membrane in Tg-BK_Ca_ mice (Figures 5A–D*)*. Protein proximity index (10) for BK_Ca_ to mitotracker and plasma membrane loaded mitochondria was 0.54 ± 0.1 (*n* = 5), and 0.2 ± 0.09 (*n* = 5) indicating a stronger correlation with mitochondria than plasma membrane. We further isolated mitochondria from the heart of WT and Tg-BK_Ca_ mice and observed mitochondrial localization of BK_Ca_ (PPI of 0.6 ± 0.1, *n* = 6 and 0.71 ± 0.2, *n* = 5, respectively, Figures 5E–M*)*. Our observations indicate that the genetic alteration of BK_Ca_ (BK^R207Q^) does not affect its cardiac distribution or its mitochondrial localization. No specific signals were observed for BK_Ca_ channels in the mitochondria of *Kcnma1*^−/−^ mice (*n* = 5) as demonstrated with immunostaining (Figures 5K–M).

## Discussion

BK_Ca_ channels are modulated by calcium, voltage, and several other cellular components, making them key pathophysiological targets. Auxiliary proteins, β and γ subunits also directly regulate the activity of BK_Ca_ channels, as well as, their sensitivity toward channel activators and blockers. One of the major breakthroughs in BK_Ca_-centric research over the past two decades is the availability of small molecular openers for BK_Ca_ channels. These small molecules and pharmacological agents have provided ample opportunities to decipher the role of BK_Ca_ channels in physiological functions. The use of several pharmacological agents including, NS1619 and NS11021, to activate BK_Ca_ has been shown to play a vital role in cardioprotection and neuroprotection from IR injury. However, due to non-specific and off-target effects of drugs (40–44), clinical application of BK_Ca_ agonists is not yet initiated. Preclinical and basic research provides sufficient evidence for a translational aspect of BK_Ca_ activators but due to limitations related to specificity and the lack of potency (40–44), concerns have been raised on the role of activation of BK_Ca_ in pathological conditions (45). The need to revisit the issue related to the role of activation of BK_Ca_ in a pathological condition such as IR injury is critical in the light of recent findings that expression of BK_Ca_ is vital for cardioprotection using global (10, 32) and cardiac-specific (17) knockout mice.

In this study, we have used transgenic mice expressing the $BK_{\text{Ca}}{}^{\text{R207Q}}$ mutant subunit driven by *Per1* locus. Per1 is ubiquitously expressed in a wide variety of tissues and in a previous study, it was shown that Tg-$BK_{\text{Ca}}{}^{\text{R207Q}}$ mice displayed overexpression of constitutively-activated BK_Ca_ in several different tissues including aorta. In adult cardiomyocytes and cardiac mitochondria isolated from Tg-BK_Ca_ mice, there were no differences observed in the localization of BK_Ca_, implying that overexpression of the $BK_{\text{Ca}}{}^{\text{R207Q}}$ mutant does not interfere with its cellular or organelle localization. We have not observed any abnormal phenotype or behavior in Tg-BK_Ca_ mice. Cardiovascular analysis using echocardiography also did not demonstrate abnormal cardiac function, in comparison to the wild-type mice. Hence, Tg-BK_Ca_ mice provide an appropriate tool to test whether overexpression and activation of BK_Ca_ play a role in cardioprotection as shown by global (10) and cardiomyocytes-specific (17) null mutant mice.

In the last two decades, several studies using pharmacological tools have suggested a protective role of BK_Ca_ from IR injury and in IPC-mediated cardioprotection and are summarized in recent reviews (1, 5). The first study to implicate BK_Ca_ in cardioprotection (i.e., improved LVDP and reduced infarction) from global IR injury was conducted using the agonist NS1619, the effects of which were blocked by paxilline (20). Improved LVDP by preconditioning with 3 μM NS1619 was confirmed by Stowe et al. (38) possibly by modulating mitochondrial ROS and Ca^2+^ concentrations. The same group has proposed that opening of BK_Ca_ elevates the level of K^+^ in the matrix of mitochondria, which is exchanged for H^+^ ion by a K^+^/H^+^ exchanger (46). Increase in the level of H^+^ in the mitochondrial matrix stabilizes mitochondrial membrane potential (ΔΨ_m_) so that unutilized electrons combine with oxygen to generate a small amount of mitochondrial oxygen radicals and other ROS, which then protects the cardiomyocytes by stimulating downstream protective signaling pathways. In addition to NS1619, studies involving other BK_Ca_ channel openers, NS11021 (14) and naringenin (47), have also demonstrated that cellular and cardioprotection from IR injury further provide strong evidence for the role of BK_Ca_ in cardioprotection. However, in primary rat cortical neurons, NS1619-preconditioning caused mitochondrial depolarization which could not be prevented by paxilline (48).

Pharmacological data using agonists and antagonists provides strong evidence for the role of BK_Ca_ in cardioprotection. However, the same set of drugs have resulted in non-specific effects in cells and organs. Paxilline, although is a specific BK_Ca_-blocker, abolished isoflurane-mediated cardioprotection in wild-type as well as *Kcnma1*^−/−^ mice^12^. NS1619 (>10 μM) is known to inhibit SERCA (49), mitochondrial respiratory chain (44, 50), and H^+^/K^+^ leak (46). These issues have largely been addressed by using low concentrations (≤10 μM) of NS1619 in conjunction with global (10) and cardiomyocytes specific (17) BK_Ca_ knockout mice. Our results using activated BK_Ca_ reiterated and further supported the studies involving global (10, 12, 32) and cardiomyocytes specific (17) knockout mice.

Interestingly, we observed an increase in the LVDP amongst a few hearts from Tg-BK_Ca_ mice exposed to IPC (4 out of 7) beyond 100% during the reperfusion stage, in comparison to the baseline. We used a constant flow approach for this experiment. We anticipate that in the presence of some degree of infarction, the remaining cardiomyocytes would contract more and beat faster to balance the pressure overload arising due to the constant flow of buffer solution to the isolated heart. An increase in LVDP and + dp/dt was also observed by Okazaki et al. after IR-injury (51). Similarly, Papanicolaou et al., also observed an increase in LDVP up to 150% in mitofusin-2 deleted hearts after 10 min of ischemia and 20 min of reperfusion *ex vivo*, using Langendorff model (52). Moreover, an increase in the LVDP is associated with an increase in the flow rate (51) in *ex vivo* IR study and BK_Ca_ activator, rottlerin, is known to increase the flow rate post *ex vivo* IR (24).

As others have shown, we too provide evidence that BK_Ca_ is exclusively present in mitochondria of adult cardiomyocytes and modulate mitochondrial function. In a recently published study, the notion of BK_Ca_ in adult cardiomyocytes playing a role in cardioprotection was confirmed, and cardioprotective role via BK_Ca_ in smooth muscle cells was ruled out by using tissue-specific knockouts (17). However, an argument regarding the role or necessity of activation of BK_Ca_ to provide cardioprotection from IR injury is still valid. Our study involving mice expressing activated BK_Ca_ without any pharmacological tools support the notion that in addition to the expression of BK_Ca_, activation of BK_Ca_ is also important in cardioprotection from IR injury. Expression of $BK_{\text{Ca}}{}^{\text{R207Q}}$ protected the heart from IR injury, as well, as improved the recovery of LVDP after IPC and IR injury. Further, we also noticed a remarkable decrease in myocardial infarction in hearts isolated from Tg-BK_Ca_ mice as compared to wild-type mice. IPC as anticipated, reduced myocardial infarction in wild-type mice which was further augmented in Tg-BK_Ca_ mice, implying that activation of BK_Ca_ can further enhance the IPC-mediated cardioprotection from IR injury. Soltysinka et al. (32) demonstrated a cause-effect relationship between “IPC-mediated cardioprotection” and “BK_Ca_” using global BK_Ca_ knockout mice. Cardioprotective property of IPC was lost in the hearts collected from BK_Ca_ knockout mice (31). Our results along with other studies using global or tissue-specific knockout mice imply that expression and activation of BK_Ca_ play an important role in cardioprotection from IR injury.

In IR injury, ROS is well-characterized to be the major player as a second messenger involved in preconditioning (53). Complex III of the electron transport chain (ETC) is the main site for ROS production, and the ROS produced is directed away from the antioxidant defenses of the mitochondrial matrix (54). However, complex I mediated ROS products are released in the mitochondrial matrix in the proximity of defense enzyme systems and is known to change the redox state of proteins present in the mitochondrial matrix, which causes a deleterious impact on cellular physiology (55). Association of K^+^ channels to mitochondrial ROS has been well-established (56, 57). Specifically, K_ATP_ channels mediate influx of K^+^ into the mitochondrial matrix resulting in a small augmentation of ROS production to induce cardioprotection (56, 57). In contrast, pharmacological tools aimed at BK_Ca_ channels indicate that the opening of BK_Ca_ reduces IR-induced large-scale ROS production whereas closing the BK_Ca_ channel increases deleterious ROS production (22, 24, 58). Recently, using cardiac-specific BK_Ca_ knockout mice, it was shown that the absence of BK_Ca_ increases ROS (17) which has been proposed earlier to regulate endoplasmic calcium release (59). Increase in ROS in the knockout mice is independent of change in expression of ROS degrading enzymes such as CuZnSOD (SOD1) and MnSOD (SOD2) (17). We have observed that activation of BK_Ca_ does not affect the ROS production at the basal levels, however, after IR injury, activation of BK_Ca_ results in a reduction in ROS production. The decline was observed for both complexes of the ETC in isolated mitochondria from Tg-BK_Ca_ mice. The other factor to be taken into account is the amount in addition to the site of production of ROS available in the cell (55). IPC reduced ROS generated by complexe I in wild-type mice which indicate that reducing the total amount of ROS can also protect the cardiac tissue.

Activation of BK_Ca_ is also known to increase the Ca^2+^ retention capacity of mitochondria (10). Our recent findings on Ca^2+^ (10), and ROS in this study, and studies from other groups have indicated that BK_Ca_-mediated cardioprotection involves an interplay between ROS, Ca^2+^ and mitochondrial permeability transition pore (mPTP) (1, 5, 22, 31, 38, 46, 60–62). One possible mechanism is an increase in Ca^2+^ retention capacity possibly by modulating a mitochondrial Ca^2+^ pump on activation of BK_Ca_, hence allowing more Ca^2+^ uptake during ischemia-reperfusion. Also, blocking BK_Ca_ channels either pharmacologically or genetically enhances ROS production, hence increasing myocardial infarction. The precise manner of how Ca^2+^ modulates ROS generation is not well-understood. However, three-dimensional conformational changes induced by Ca^2+^ in the ETC complexes, such as changes in complex IV have been reported (63, 64). Direct addition of BK_Ca_ channel activator in isolated mitochondrial preparation results in depolarization (5, 46) of mitochondrial membrane potential which made it difficult to study mitochondrial BK_Ca_ channels in isolated preparations. However, the use of genetic approaches clearly indicates that partial activation (Tg-BK_Ca_) does not change mitochondrial ROS generation under physiological conditions. During stress, the increase in Ca^2+^ influx could further increase the open probability of the channel which could result in a reduction of ROS as observed in our studies. Since the decrease in ROS levels has been associated with cardioprotection from IR injury (65, 66), we anticipate that activation of BK_Ca_ reduces deleterious ROS production, hence decreases Ca^2+^ release from endoplasmic reticulum and reduction in an influx of Ca^2+^ to the matrix and prevents Ca^2+^ overload in mitochondria (59). This complex interplay between Ca^2+^ and ROS is known to result in apoptosis, possibly by opening the mPTP. Hence, activation of BK_Ca_ can be linked to delay in the formation and/or closing of mPTP.

In summary, our study implicates overexpression of activated BK_Ca_ in cardioprotection against IR injury, and cardioprotection is mediated, in part, by decreasing deleterious mitochondrial ROS generation.

## Limitations of the Study

Even though, our study does not involve pharmacological tools, there are several limitations which should be mentioned. The Tg-BK_Ca_ mice are not homozygous but are generated in the background of wild-type mice as the homozygous mouse is embryonic lethal (8). The animals are phenotypically normal. This finding is important as usage of heterozygous mice suggests that partial activation of BK_Ca_ is sufficient for cardioprotection and reduction of mitochondrial ROS. The gain-of-function BK_Ca_ is present in all the cells under *Per1* locus. Since Per1 is present in most cells types, the effect we observed could also arise from non-cardiomyocytes. Non-cardiomyocyte cells such as cardiac neurons are known to play a role in cardioprotection as well as cellular protection as reported earlier (12). Our study does not rule out the role of non-cardiomyocyte BK_Ca_ in cardioprotection from IR injury. Our *ex vivo* approach using the Langendorff method partially rules out non-cardiac BK_Ca_ as the heart is excised and isolated from other organs during the experiment. Since hearts were not paced at a constant rate, ± dp/dt may not be a good index of cardiac function. Therefore, we did not report ± dp/dt. We further isolated mitochondria and measured ROS levels with and without IR. Our results also indicate that BK_Ca_ channel activation can modulate mitochondrial ROS levels. We have observed statistical significance in between WT and Tg-BK_Ca_ IR for LVDP, infarction, and ROS production but not in WT IR vs. IPC when one-way ANOVA followed by Tukey's multiple comparison tests was used. This could be due to the shorter duration of reperfusion (30 min).

Tg-BK_Ca_ construct is generated on DEC splice variant (8) which is known to facilitate localization of BK_Ca_ to mitochondria (10), and we did not observe any change in localization of BK_Ca_ in cardiomyocytes or isolated mitochondria. Therefore, we corroborate earlier findings involving pharmacological and global, as well as cardiac-specific null mutant mice, in addition to the expression of BK_Ca_, activation of BK_Ca_ plays an important role in cardioprotection from IR injury.

## Ethics Statement

The study was carried out in accordance with the recommendations from National Institute of Health. All protocols involving animals were approved by the Drexel University College of Medicine and the Ohio State University IACUC.

## Author Contributions

SKG, DP, ATH, KS, PK, SGR, MK, and HS performed the research, analyzed data and wrote the manuscript. ALM generated Tg-BK and BK mutant mice.

### Conflict of Interest Statement

The authors declare that the research was conducted in the absence of any commercial or financial relationships that could be construed as a potential conflict of interest.

## References

[1] Singh H Stefani E Toro L. Intracellular BK(Ca) (iBK(Ca)) channels. J Physiol. (2012) 590:5937–47. 10.1113/jphysiol.2011.215533 22930268 PMC3530108

[2] Toro L Li M Zhang Z Singh H Wu Y Stefani E. MaxiK channel and cell signalling. Pflugers Arch. (2012) 466:875–86. 10.1007/s00424-013-1359-0 24077696 PMC3969412

[3] Hite RK Tao X MacKinnon R. Structural basis for gating the high-conductance Ca^2+^-activated K^+^ channel. Nature (2017) 541:52–7. 10.1038/nature20775 27974801 PMC5513477

[4] Salkoff L Butler A Ferreira G Santi C Wei A. High-conductance potassium channels of the SLO family. Nat Rev Neurosci. (2006) 7:921–31. 10.1038/nrn1992 17115074

[5] Balderas E Zhang J Stefani E Toro L. Mitochondrial BKCa channel. Front Physiol. (2015) 6:104. 10.3389/fphys.2015.00104 25873902 PMC4379900

[6] Li B Jie W Huang L Wei P Li S Luo Z. Nuclear BK channels regulate gene expression via the control of nuclear calcium signaling. Nat Neurosci. (2014) 17:1055–63. 10.1038/nn.3744 24952642 PMC4115017

[7] Meredith AL Thorneloe KS Werner ME Nelson MT Aldrich RW. Overactive bladder and incontinence in the absence of the BK large conductance Ca^2+^-activated K^+^ channel. J Biol Chem. (2004) 279:36746–52. 10.1074/jbc.M405621200 15184377

[8] Montgomery JR Meredith AL. Genetic activation of BK currents in vivo generates bidirectional effects on neuronal excitability. Proc Natl Acad Sci USA. (2012) 109:18997–9002. 10.1073/pnas.1205573109 23112153 PMC3503162

[9] Singh H Li M Hall L Chen S Sukur S Lu R. MaxiK channel interactome reveals its interaction with GABA transporter 3 and heat shock protein 60 in the mammalian brain. Neuroscience (2016) 317:76–107. 10.1016/j.neuroscience.2015.12.058 26772433 PMC4737998

[10] Singh H Lu R Bopassa JC Meredith AL Stefani E Toro L mitoBKCa is encoded by the Kcnma1 gene, and a splicing sequence defines its mitochondrial location. Proc Natl Acad Sci USA. (2013) 110:10836–41. 10.1073/pnas.1302028110 23754429 PMC3696804

[11] Shi Y Jiang MT Su J Hutchins W Konorev E Baker JE. Mitochondrial big conductance KCa channel and cardioprotection in infant rabbit heart. J Cardiovasc Pharmacol. (2007) 50:497–502. 10.1097/FJC.0b013e318137991d 18030058

[12] Wojtovich AP Nadtochiy SM Urciuoli WR Smith CO Grunnet M. A non-cardiomyocyte autonomous mechanism of cardioprotection involving the SLO1 BK channel. Peer J. (2013) 1:e48. 10.7717/peerj.48 23638385 PMC3628382

[13] Shintani Y Node K Asanuma H Sanada S Takashima S Asano Y. Opening of Ca^2+^-activated K^+^ channels is involved in ischemic preconditioning in canine hearts. J Mol Cell Cardiol. (2004) 37:1213–8. 10.1016/j.yjmcc.2004.09.012 15572051

[14] Bentzen BH Osadchii O Jespersen T Hansen RS Olesen SP Grunnet M Activation of big conductance Ca^2+^-activated K^+^ channels (BK) protects the heart against ischemia-reperfusion injury. Pflugers Arch. (2009) 457:979–88. 10.1007/s00424-008-0583-5 18762970

[15] Behmenburg F Dorsch M Huhn R Mally D Heinen A Hollmann MW. Impact of mitochondrial Ca^2+^-sensitive potassium (mBKCa) channels in sildenafil-induced cardioprotection in rats. PLoS ONE (2015) 10:e0144737. 10.1371/journal.pone.0144737 26671662 PMC4684397

[16] Patel NH Johannesen J Shah K Goswami SK Patel NJ Ponnalagu D. Inhibition of BKCa negatively alters cardiovascular function. Physiol Rep. (2018) 6:e13748. 10.14814/phy2.13748 29932499 PMC6014461

[17] Frankenreiter S Bednarczyk P Kniess A Bork NI Straubinger J Koprowski P. cGMP-elevating compounds and ischemic conditioning provide cardioprotection against ischemia and reperfusion injury via cardiomyocyte-specific BK channels. Circulation (2017) 136:2337–55. 10.1161/CIRCULATIONAHA.117.028723 29051185

[18] Liao Y Kristiansen AM Oksvold CP Tuvnes FA Gu N Rundén-Pran E. Neuronal Ca^2+^-activated K^+^ channels limit brain infarction and promote survival. PLoS ONE (2010) 5:e15601. 10.1371/journal.pone.0015601 21209897 PMC3012709

[19] Dai H Wang M Patel PN Kalogeris T Liu Y Durante W. Preconditioning with the BKCa channel activator NS-1619 prevents ischemia-reperfusion-induced inflammation and mucosal barrier dysfunction: roles for ROS and heme oxygenase-1. Am J Physiol Heart Circ Physiol. (2017) 313:H988–99. 10.1152/ajpheart.00620.2016 28822969 PMC5792206

[20] Xu W Liu Y Wang S McDonald T Van Eyk JE Sidor A. Cytoprotective role of Ca^2+^- activated K^+^ channels in the cardiac inner mitochondrial membrane. Science (2002) 298:1029–33. 10.1126/science.1074360 12411707

[21] Kulawiak B Kudin AP Szewczyk A Kunz WS. BK channel openers inhibit ROS production of isolated rat brain mitochondria. Exp Neurol. (2008) 212:543–7. 10.1016/j.expneurol.2008.05.004 18572168

[22] Heinen A Aldakkak M Stowe DF Rhodes SS Riess ML Varadarajan SG. Reverse electron flow-induced ROS production is attenuated by activation of mitochondrial Ca^2+^-sensitive K^+^ channels. Am J Physiol Heart Circ Physiol. (2007) 293:H1400–7. 10.1152/ajpheart.00198.2007 17513497

[23] Facundo HT Fornazari M Kowaltowski AJ. Tissue protection mediated by mitochondrial K^+^ channels. Biochim Biophys Acta (2006) 1762:202–12. 10.1016/j.bbadis.2005.06.003 16026967

[24] Cordeiro B Terentyev D Clements RT. BKCa channel activation increases cardiac contractile recovery following hypothermic ischemia/reperfusion. Am J Physiol Heart Circ Physiol. (2015) 309:H625–33. 10.1152/ajpheart.00818.2014 26071546 PMC4537936

[25] Starkov AA. “Mild” uncoupling of mitochondria. Biosci Rep. (1997) 17:273–9. 9337482 10.1023/a:1027380527769

[26] Szewczyk A Jarmuszkiewicz W Kunz WS. Mitochondrial potassium channels. IUBMB Life (2009) 61:134–43. 10.1002/iub.155 19165895

[27] Xi L Hess ML Kukreja RC. Ischemic preconditioning in isolated perfused mouse heart: reduction in infarct size without improvement of post-ischemic ventricular function. Mol Cell Biochem. (1998) 186:69–77. 9774187

[28] Kohut A Patel N Singh H. Comprehensive echocardiography assessment of the right ventricle in murine models. J Cardiovasc Ultrasound. (2016) 24:229–38. 10.4250/jcu.2016.24.3.229 27721954 PMC5050312

[29] Singh H Lu R Rodríguez PF Wu Y Bopassa JC Stefani E. Visualization and quantification of cardiac mitochondrial protein clusters with STED microscopy. Mitochondrion (2012) 12:230–6. 10.1016/j.mito.2011.09.004 21982778 PMC3258335

[30] Sato T Saito T Saegusa N Nakaya H. Mitochondrial Ca^2+^-activated K^+^ channels in cardiac myocytes: a mechanism of the cardioprotective effect and modulation by protein kinase A. Circulation (2005) 111:198–203. 10.1161/01.CIR.0000151099.15706.B1 15623543

[31] Stumpner J Lange M Beck A Smul TM Lotz CA Kehl F. Desflurane-induced post-conditioning against myocardial infarction is mediated by calcium-activated potassium channels: role of the mitochondrial permeability transition pore. Br J Anaesth. (2012) 108:594–601. 10.1093/bja/aer496 22315330

[32] Soltysinska E Bentzen BH Barthmes M Hattel H Thrush AB Harper ME. KCNMA1 encoded cardiac BK channels afford protection against ischemia-reperfusion injury. PLoS ONE (2014) 9:e103402. 10.1371/journal.pone.0103402 25072914 PMC4114839

[33] Díaz L Meera P Amigo J Stefani E Alvarez O Toro L. Role of the S4 segment in a voltage-dependent calcium-sensitive potassium (hSlo) channel. J Biol Chem. (1998) 273:32430–6. 9829973 10.1074/jbc.273.49.32430

[34] Cao CM Xia Q Gao Q Chen M Wong TM. Calcium-activated potassium channel triggers cardioprotection of ischemic preconditioning. J Pharmacol Exp Ther. (2005) 312:644–50. 10.1124/jpet.104.074476 15345753

[35] Garlid AO Jaburek M Jacobs JP Garlid KD. Mitochondrial reactive oxygen species: which ROS signals cardioprotection? Am J Physiol Heart Circul Physiol. (2013) 305:H960–8. 10.1152/ajpheart.00858.2012 23913710 PMC3798754

[36] Tang XD Garcia ML Heinemann SH Hoshi T. Reactive oxygen species impair Slo1 BK channel function by altering cysteine-mediated calcium sensing. Nat Struct Mol Biol. (2004) 11:171–8. 10.1038/nsmb725 14745441

[37] Gaifullina AS Yakovlev AV Mustafina AN Weiger TM Hermann A Sitdikova GF. Homocysteine augments BK channel activity and decreases exocytosis of secretory granules in rat GH3 cells. FEBS Lett. (2016) 590:3375–84. 10.1002/1873-3468.12381 27586872

[38] Stowe DF Aldakkak M Camara AK Riess ML Heinen A Varadarajan SG. Cardiac mitochondrial preconditioning by Big Ca^2+^-sensitive K^+^ channel opening requires superoxide radical generation. Am J Physiol Heart Circ Physiol. (2016) 290:H434–40. 10.1152/ajpheart.00763.2005 16126810

[39] Stowe DF Yang M Heisner JS Camara AKS. Endogenous and agonist-induced opening of mitochondrial big versus small Ca^2+^-sensitive K^+^ channels on cardiac cell and mitochondrial protection. J Cardiovasc Pharmacol. (2017) 70:314–28. 10.1097/FJC.0000000000000524 28777255 PMC5726766

[40] Saleh SN Angermann JE Sones WR Leblanc N Greenwood IA Stimulation of Ca^2+^-gated Cl^−^ currents by the calcium-dependent K^+^ channel modulators NS1619 [1,3-dihydro-1-[2-hydroxy-5-(trifluoromethyl)phenyl]-5-(trifluoromethyl)-2H-benzi midazol-2-one] and isopimaric acid. J Pharmacol Exp Ther. (2007) 321:1075–84. 10.1124/jpet.106.118786 17347326

[41] Park WS Kang SH Son YK Kim N Ko JH Kim HK. The mitochondrial Ca^2+^-activated K^+^ channel activator, NS 1619 inhibits L-type Ca^2+^ channels in rat ventricular myocytes. Biochem Biophys Res Commun. (2007) 362:31–6. 10.1016/j.bbrc.2007.07.057 17698036

[42] Olesen SP Munch E Moldt P Drejer J Selective activation of Ca^2+^-dependent K^+^ channels by novel benzimidazolone. Eur J Pharmacol. (1994) 251:53–9. 8137869 10.1016/0014-2999(94)90442-1

[43] Holland M Langton PD Standen NB Boyle JP. Effects of the BKCa channel activator, NS1619, on rat cerebral artery smooth muscle. Br J Pharmacol. (1996) 117:119–29. 8825352 10.1111/j.1476-5381.1996.tb15163.x PMC1909362

[44] Cancherini DV Queliconi BB Kowaltowski AJ. Pharmacological and physiological stimuli do not promote Ca^2+^-sensitive K^+^ channel activity in isolated heart mitochondria. Cardiovasc Res. (2007) 73:720–8. 10.1016/j.cardiores.2006.11.035 17208207

[45] Bentzen BH Olesen SP Ronn LC Grunnet M. BK channel activators and their therapeutic perspectives. Front Physiol. (2014) 5:389. 10.3389/fphys.2014.00389 25346695 PMC4191079

[46] Aldakkak M Stowe DF Cheng Q Kwok WM Camara AK. Mitochondrial matrix K^+^ flux independent of large-conductance Ca^2+^-activated K^+^ channel opening. Am J Physiol Cell Physiol. (2010) 298:C530–41. 10.1152/ajpcell.00468.2009 20053924 PMC2838564

[47] Testai L Martelli A Marino A D'Antongiovanni V Ciregia F Giusti L. The activation of mitochondrial BK potassium channels contributes to the protective effects of naringenin against myocardial ischemia/reperfusion injury. Biochem Pharmacol. (2013) 85:1634–43. 10.1016/j.bcp.2013.03.018 23567997

[48] Gáspár T Domoki F Lenti L Katakam PV Snipes JA Bari F. Immediate neuronal preconditioning by NS1619. Brain Res. (2009) 1285:196–207. 10.1016/j.brainres.2009.06.008 19523929 PMC2744349

[49] Wrzosek A. The potassium channel opener NS1619 modulates calcium homeostasis in muscle cells by inhibiting SERCA. Cell Calcium (2014) 56:14–24. 10.1016/j.ceca.2014.03.005 24813114

[50] Kicinska A Szewczyk A. Large-conductance potassium cation channel opener NS1619 inhibits cardiac mitochondria respiratory chain. Toxicol Mech Methods (1996) 14:59–61. 10.1080/15376520490257482 20021124

[51] Okazaki Y Cao ZL Ohtsubo S Hamada M Naito K Rikitake K. Leukocyte-depleted reperfusion after long cardioplegic arrest attenuates ischemia-reperfusion injury of the coronary endothelium and myocardium in rabbit hearts. Eur J Cardiothorac Surg. (2000) 18:90–7. 10.1016/s1010-7940(00)00436-x 10869946

[52] Papanicolaou KN Khairallah RJ Ngoh GA Chikando A Luptak I O'Shea KM. Mitofusin-2 maintains mitochondrial structure and contributes to stress-induced permeability transition in cardiac myocytes. Mol Cell Biol. (2011) 31:1309–28. 10.1128/MCB.00911-10 21245373 PMC3067905

[53] Garlid KD Costa AD Quinlan CL Pierre SV Dos Santos P. Cardioprotective signaling to mitochondria. J Mol Cell Cardiol. (2009) 46:858–66. 10.1016/j.yjmcc.2008.11.019 19118560 PMC2683183

[54] Chen Q Vazquez EJ Moghaddas S Hoppel CL Lesnefsky EJ. Production of reactive oxygen species by mitochondria: central role of complex III. J Biol Chem. (2003) 278:36027–31. 10.1074/jbc.M304854200 12840017

[55] Sanz A. Mitochondrial reactive oxygen species: do they extend or shorten animal lifespan? Biochim Biophys Acta (2016) 1857:1116–26. 10.1016/j.bbabio.2016.03.018 26997500

[56] Garlid KD Paucek P Yarov-Yarovoy V Sun X Schindler PA. The mitochondrial KATP channel as a receptor for potassium channel openers. J Biol Chem. (1996) 271:8796–9. 8621517 10.1074/jbc.271.15.8796

[57] Andrukhiv A Costa AD West IC Garlid KD. Opening mitoKATP increases superoxide generation from complex I of the electron transport chain. Am J Physiol Heart Circ Physiol. (2006) 291:H2067–74. 10.1152/ajpheart.00272.2006 16798828

[58] Stowe DF Camara AK. Mitochondrial reactive oxygen species production in excitable cells: modulators of mitochondrial and cell function. Antioxid Redox Signal. (2009) 11:1373–414. 10.1089/ARS.2008.2331 19187004 PMC2842133

[59] Booth DM Enyedi B Geiszt M Varnai P Hajnoczky G. Redox nanodomains are induced by and control calcium signaling at the ER-mitochondrial interface. Mol Cell (2016) 63:240–8. 10.1016/j.molcel.2016.05.040 27397688 PMC4998968

[60] Aldakkak M Camara AK Heisner JS Yang M Stowe DF. Ranolazine reduces Ca^2+^ overload and oxidative stress and improves mitochondrial integrity to protect against ischemia reperfusion injury in isolated hearts. Pharmacol Res. (2011) 64:381–92. 10.1016/j.phrs.2011.06.018 21741479 PMC3233383

[61] Aldakkak M Stowe DF Chen Q Lesnefsky EJ Camara AK. Inhibited mitochondrial respiration by amobarbital during cardiac ischaemia improves redox state and reduces matrix Ca^2+^ overload and ROS release. Cardiovasc Res. (2008) 77:406–15. 10.1016/j.cardiores.2007.08.008 17900548

[62] Heinen A Camara AK Aldakkak M Rhodes SS Riess ML Stowe DF. Mitochondrial Ca^2+^-induced K^+^ influx increases respiration and enhances ROS production while maintaining membrane potential. Am J Physiol Cell Physiol. (2007) 292:C148–56. 10.1152/ajpcell.00215.2006 16870831

[63] Wikstrom M Saari H. A spectral shift in cytochrome a induced by calcium ions. Biochim Biophys Acta (1975) 408:170–9. 811258 10.1016/0005-2728(75)90009-2

[64] Brookes PS Yoon Y Robotham JL Anders MW Sheu SS. Calcium, ATP, and ROS: a mitochondrial love-hate triangle. Am J Physiol Cell Physiol. (2004) 287:C817–33. 10.1152/ajpcell.00139.2004 15355853

[65] Ponnalagu D Singh H. Anion channels of mitochondria. Handb Exp Pharmacol. (2017) 240:71–101. 10.1007/164_2016_39 27783269 PMC5855116

[66] Gururaja Rao S Ponnalagu D Patel NJ Singh H. Three decades of chloride intracellular channel proteins: from organelle to organ physiology. Curr Protoc Pharmacol. (2018) 80:1–17. 10.1002/cpph.36 30040212 PMC6060641

